# Utilization of social franchising in family planning services: a Pakistan perspective

**DOI:** 10.3389/fgwh.2024.1376374

**Published:** 2024-05-17

**Authors:** Nayab, Taimoor Ahmad, Areesh Fatmee, Ibtisam Sajjad, Zona Usmani, Ayesha Khan, Sara Shahzad, Adnan Ahmad Khan

**Affiliations:** ^1^ Program, Research and Development Solutions (RADS), Islamabad, Pakistan; ^2^ Urban Impact Lab, Akhter Hameed Khan Foundation, Islamabad, Pakistan; ^3^ University of Cambridge, Cambridge, United Kingdom; ^4^ Ministry of National Health Services, Regulations and Coordination (MoNHSRC), Islamabad, Pakistan

**Keywords:** family planning, social franchising, COVID-19, volume of clients, volume of products, privacy of clients, clients of family planning

## Abstract

**Introduction:**

Pakistan's private sector caters to around 65% of family planning users. Private sector family planning was promoted in the Delivering Accelerated Family Planning in Pakistan (DAFPAK) program by UK's Foreign, Commonwealth & Development Office (FCDO) in 2019. We use data from DAFPAK to analyze the clientele and products distributed by two major NGOs, Marie Stopes Society (MSS) and DKT Pakistan, that support private providers in Pakistan. We also examined the effect of COVID-19 on client visits and contraceptives uptake at private facilities in Pakistan.

**Methods:**

DAFPAK used field validation surveys to analyze the volume of clients and products of 639 private facilities across three provinces (Punjab, KPK and Balochistan) of Pakistan. The data was collected in two phases (February 2020 and 2021) using multi-stage cluster sampling at 95% confidence level. Using a generalized negative binomial regression, facility-level characteristics and impact of COVID-19 was analyzed with the volume of clients and products given out at 95% confidence interval alongside descriptive analysis.

**Results:**

DKT facilities covered 53% of the sample while MSS covered 47%, with 72% facilities in the rural areas. Average facility existence duration is 87 months (7.25 years). While the average experience of the facility staff is 52 months (4.33 years). MSS is serving more clients as compared to DKT during both phase 1 (IRR: 3.15; 95% CI: 2.74, 3.61) and phase 2 (IRR: 2.11; 95% CI: 1.79, 2.49). Similarly, MSS had a greater volume of products given out in both phases 1 (IRR: 1.89; 95% CI: 1.51, 2.38) and phase 2 (IRR: 2.57; 95% CI: 2.09, 3.14). In both phases, client visits and product distribution decreased when client privacy is invaded (IRR: 0.74; 95% CI: 0.67, 0.82 – phase 1) and (IRR: 0.83; 95% CI: 0.72, 0.97 – phase 2). Lastly, during COVID-19, products distribution decreased by a factor of 0.84 (IRR: 0.84; 95% CI: 0.72, 0.97) but client visits remain unaffected.

**Conclusion:**

Overall, clientele is low for all facilities. At a facility, privacy is a determinant of client visits and products given out per visit. Transiently, during COVID-19, client volumes decreased, with a shift from oral pills to condoms and emergency contraceptive pills.

## Introduction

Pakistan is the world's fifth most populous country of 241 million people. It had committed at the London Family Planning summit 2012 to raise its Contraceptive Prevalence Rate (CPR) to 55% by 2020 (1). This was later modified to 50% by 2025% and 60% by 2030 and the commitment was institutionalized nationally as “Recommendations of the Council of Common Interests” (CCI) and Pakistan's FP2030 pledge (2, 3). Despite political commitment, allocated budgets, and large-scale family planning (FP) programs, the national contraceptive prevalence rate (CPR) has remained in the 30%–35% range since 2007 (3–6), and Pakistan's population growth rate increased from 2.4% (Census 2017) to 2.55% (Census 2023) (7, 8).

Approximately 36%–65% of family planning services in low and middle-income countries (LMIC) are offered by private sector providers through social franchising initiatives (9). In Pakistan, the private sector plays a dominant role, albeit, mainly through social marketing outlets such as pharmacies, markets, shops, etc., that serve 54% of users that avail FP each year, and is expanding relatively slowly (3–6). On the other hand, socially franchised private clinics serve another 11% (3, 10, 11). Government services, including lady health workers and clinics, serve around 35% of users in a year. Among these, the outreach of LHW may have maximized and is not expanding (12, 13). Both private or public health facilities remain underutilized and therefore potentially have the most room for expansion (14).

Nearly all private FP facilities are supported by NGOs through the social franchising approach where private healthcare providers are included into networks where they are shared in a brand that gives them credibility in the communities they operate. In return, this branding allows the supporting NGO to assure quality of services and products that engenders trust among clients. The approach allows flexibility in services and method choices, and adaptability to the local context and needs in order to promote sustainability, albeit often with grant supported subsidies for providers (15, 16). The concept is well established, with demonstrated success. For example, programs from Pakistan showed an 11% increase in IUDs use (17), while 22% rise in Long Acting Reversible Contraception (LARC) utilization was observed in Kenya through social franchising programs (18).

Given the potential importance of private clinics in scaling up FP services, factors that affect facility utilization must be studied. Facility-level factors that impact client satisfaction include the lack of privacy (19), limited autonomy (20), inadequate communication (21), and poor sanitation (22). Inadequate quality of care may result in clients not returning for future services and premature discontinuation of contraceptives (23). Secular events such as large lockdowns that happened during COVID-19 pandemic, can reduce clinic attendance (24).

COVID-19 caused major disruptions from the illness itself, as well as from lockdowns instituted to curb infections. Pakistan saw five waves during the COVID-19 pandemic over two years (Khan et al, under review). Health services, including family planning, were disrupted, due to lockdowns when either clinics or outreach closed or when supplies could not be delivered, or because people opted to stay home. The United Nations Population Fund (UNFPA) estimated that access to FP services would be constrained due to COVID-19 for 12 million women in lower and middle income countries (LMIC), resulting in 1.4 million unintended pregnancies (25). In Pakistan, the health ministry records suggest that services resumed very quickly after the first quarter. A study of FP services in recent years must account for the impact of COVID-19 on these services.

The current study is the first exploration of volume of clients and the factors that influence such utilization, including the impact of COVID-19 disruptions, from two of Pakistan's largest private-sector clinic providers to understand healthcare utilization in the private sector. We used data from the Delivering Accelerated Family Planning in Pakistan (DAFPAK) program (2019–2025) by UK's Foreign, Commonwealth & Development Office (FCDO). DAFPAK was designed to increase access and quality of FP services through the public and private sectors, and is the largest donor funded FP program in Pakistan. The program funded social franchising implementing partners Marie Stopes Society (MSS) and DKT International to support private sector clinic based services (26).

## Data and methodology

The study aims to evaluate the volume of clientele utilizing family planning services and examine the influence of facility-level characteristics, as well as the disruptions caused by COVID-19, on the utilization of these services. To attain this, we conducted a generalized negative binomial regression analysis.

### Sample size data

Third-party validation and monitoring (TPV&M) of DAFPAK provided FCDO with insights, regular updates and assurance on the delivery, progress, and impact of the project. TPV&M collated quarterly reports and validated the results through six monthly field validation of implementing partners' (IPs) performance through provider assessments, product verification, facility/outlet verification, and training verification for private service providers through field surveys.

This study uses survey data collected during DAFPAK's six-monthly field validations survey. The analysis includes only those facilities that were active and serving clients at the time of the survey. Facilities not serving clients were excluded the data was collected in two phases. In the first phase, independent six-monthly field validation took place during February 2020 and validated the IPs results reported for the period July to December 2019. While in the second phase, independent six-monthly field validation was carried out during February 2021. The second phase reflects data collected for services rendered while the COVID-19 epidemic was extant.

We used a multi-stage cluster random sampling methodology, ensuring representation of all IPs service delivery modalities across DAFPAK implementation districts (which were treated as clusters for sampling). Using a UNICEF MICS 3 sample size calculator, and assuming a design effect of 1.5 with a marginal error of 95% confidence interval at 0.12 and allowing for up to 10% refusals. Data collection was done through electronic tablets using SurveyCTO.

The six-monthly data collection covered 324 and 355 facilities in the first and second phase of the program respectively. Facilities which did not serve any client were dropped from the analysis. In phase one and two, 23 and 17 facilities were dropped. Therefore, the final data included 301 and 338 facilities in phase one and two.

In phase 1, a total of 301 facilities (DKT = 163 and MSS = 138) were covered from 20 districts in 3 provinces. Eleven districts were from Khyber Pakhtunkhwa (KPK), 8 from Punjab, and 1 from Balochistan. In phase 2, 338 facilities (DKT = 178 and MSS = 160) were selected from a total of 15 districts; 9 from KPK, 5 from Punjab, and 1 from Balochistan. In total 639 facilities were spread across two time periods.

### Variables

We have used count data representing the number of clients and products dispatched by each facility along with their characteristic variables.

### Dependent variables

The number of clients visiting a facility for each family planning method was summed up for all facilities to generate the variable for *volume of clients* at the facility level for the last month. A similar variable for *volume of products* given out was obtained by adding the products given out by each facility in the sample for the last month. The volume of clients and products are used as the dependent variables in the regression analysis.

### Independent variables

*Facility type* is a binary variable representing the type of family planning facility. The variable is given a value of 0 if the facility is operated by DKT and a value of 1 for MSS. *Duration of facility* is a continuous variable showing the duration in months since the facility has started its operation.

The variable *region* is a binary variable depicting if the facility is operating in an urban or rural area. A value of 1 is assigned if the facility is located in the urban settlement and 0 otherwise. *Work experience* is a continuous variable measured in terms of months where the minimum number of months a worker has worked with a facility is 2 months and the highest is 240 months. Work experience of service-providing staff can influence FP method uptake (26).

Moreover, variables presenting other infrastructural characteristics like availability of *logo, washroom, and information, education, and communication (IEC) material like booklets, brochures, and charts* may affect client satisfaction and visits to a facility. These are used as binary variables (No = 0 and Yes = 1) for this analysis. Since family planning is presumed to be dealt with privacy and maintaining privacy at facility center influences client's satisfaction and uptake of contraceptive (27, 28). A binary variable (overhearing conversation in the counseling area) is used as a proxy for *privacy invasion*. The variable takes on a value of 1 if people in the waiting room can overhear the conversation happening in the counselling area and 0 otherwise.

Lastly, COVID-19 is a binary variable taking on a value of 1 if the data collection was carried out during the COVID-19 time period and 0 otherwise. The second phase (second six monthly) of data collection took place during the COVID-19.

### Statistical analysis

Preliminary cross-tabulations were performed to observe the empirical differences in the volume of clients and products between MSS and DKT. Based on these results, we further analyzed the association between the facility level characteristics and COVID-19 on the volume of clients and products.

Both datasets consist of count data collected at the facility level. The data was over-dispersed for outcome variables (Table 1). Hence, instead of an ordinary least square (OLS) or Poisson regression, a generalized negative binomial regression is used for this analysis. A negative binomial regression is a special type of regression that is used to determine the association between confounding variables on a count outcome variable that has over-dispersed data (13).

**Table 1:** Summary statistics of variables (February 2020 and February 2021).

Variables	2019–2020				2020–2021			
	DKT (*N* = 163)		MSS (*N* = 138)		DKT (*N* = 178)		MSS (*N* = 160)	
	*N*	Mean/%^a^	*N*	Mean/%^a^	*N*	Mean/%^a^	*N*	Mean/%^a^
Volume of clients	163	22	138	71	178	27	160	58
Volume of products	163	56	138	108	178	42	160	110
Region								
Urban	48	29%	38	28%	53	30%	39	24%
Rural	115	71%	100	72%	125	70%	121	76%
Duration of facility (Months)	163	74	138	77	178	96	160	100
Work experience (Months)	163	51	138	45	178	60	160	49
NGO logo displayed								
No	14	9%	5	4%	20	11%	12	7%
Yes	149	91%	133	96%	158	89%	148	93%
Privacy invasion								
No	72	44%	84	61%	71	40%	116	72%
Yes	91	56%	54	39%	107	60%	44	28%
Washroom								
No	6	4%	1	1%	7	4%	0	0%
Yes	157	96%	1,378	99%	171	96%	160	100%
IEC brochures								
No	43	28%	7	5%	30	17%	18	11%
Yes	112	72%	129	95%	148	83%	142	89%
IEC booklet								
No	60	39%	37	27%	54	30%	24	15%
Yes	95	61%	99	73%	124	70%	136	85%
IEC chart								
No	19	12%	11	8%	13	7%	3	2%
Yes	136	88%	125	92%	165	93%	157	98%

^a^ For continuous variables, we have computed the mean and percentages for the categorical variables.

The negative binomial regression equation used is as follows:

$$\begin{array}{rl} & \mathrm{Volume}\;\mathrm{of}\;\mathrm{Client}/\mathrm{Product}s_{\mathrm{it}\;}=\beta_{1}+\beta_{2}\;\mathrm{Regio}n_{\mathrm{it}}+\beta_{3}\;\mathrm{Duratio}n_{\mathrm{it}}\\ & \;+\beta_{4}\;\mathrm{Experienc}e_{\mathrm{it}}+\beta_{5}\;\mathrm{Log}o_{\mathrm{it}}+\beta_{6}\;\mathrm{Privac}y_{\mathrm{it}}+\beta_{7}\;\mathrm{Washroo}m_{\mathrm{it}}\\ & \;+\beta_{8}\;\mathrm{IEE}\;\mathrm{Brochur}e_{\mathrm{it}}+\beta_{9}\;\mathrm{IEE}\;\mathrm{Bookle}t_{\mathrm{it}}+\beta_{10}\;\mathrm{IEE}\;\mathrm{Char}t_{\mathrm{it}}\\ & \;+\beta_{11}\;\mathrm{COVID}\text{-}19_{t}+\varepsilon_{\mathrm{it}} \end{array}$$

Generalized negative binomial regression is used to identify association between the type of facility (DKT/MSS) and region with volume of clients/products for the first and second phase separately. We have also included other facility-level characteristics like duration of facility, work experience of staff, availability of logo, washroom, IEC material and privacy that may affect the volume of clients and products.

Generalized negative binomial regression was also used to assess the impact of COVID-19 on volume of clients and products. Since first phase covers the pre COVID-19 time period and the second phase was initiated during the COVID-19. The datasets for both periods were appended and a binary variable for COVID-19 was generated (pre-COVID-19 = 0 and COVID-19 = 1) to incorporate the COVID-19 effect. Each regression was run separately for volume of clients and products.

Lastly, in order to analyze the impact of COVID-19 on client visits and product uptake of each method, generalized negative binomial regressions were run for each method type. The analysis was performed using the statistical software STATA 17.

## Results

**Figure 1:**
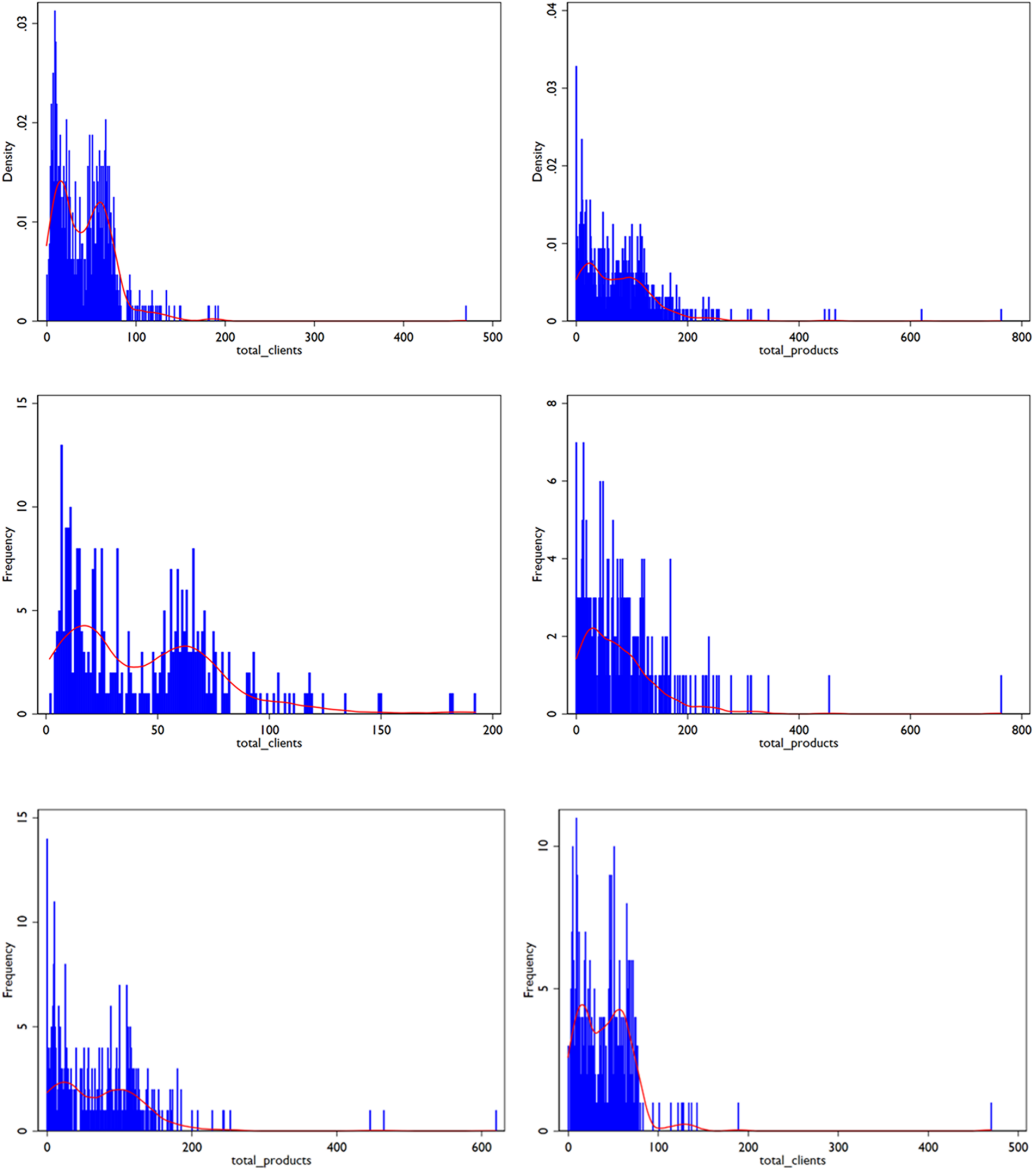
Density distribution of Volume of Clients and Products (Both Phases).

In the preliminary analysis, histograms of the total volume of clients and products were generated to assess the distribution of the data (Figure 1). Both distributions showed a right-skewed pattern. The skewness and kurtosis tests indicate that both volume of clients (*p*-value 0.000) and products (*p*-value 0.000) significantly deviate from normal distribution. The study included 163 DKT and 138 MSS facilities in phase one, and 178 DKT and 160 MSS facilities in phase two (Table 1). Most facilities (72%) were situated in the rural areas. On average each facility had been in existence for 87 months (7.25 years) and the average experience of the facility staff was around 52 months (4.33 years).

MSS served 9,784 and 9,267 clients per facility in phases 1 and 2 (Table 2). In phase 1, MSS facilities served an average of 71 clients per month, compared to 22 by DKT. During the second phase, MSS served an average of 58 clients per month, compared to 27 by DKT. MSS facilities served more clients with condoms and intra-uterine contraceptive device (IUCD) services, while DKT served more clients for the combined oral contraceptive (CoC), emergency contraceptive (EC) pills and injections. Notably, the average clientele per month for MSS dropped by 13 per month during the second phase, while it increased for DKT by 5.

**Table 2:** Volume of clients served by facility type and products in the last month.

Service	2019–2020				2020–2021			
	DKT		MSS		DKT		MSS	
	*N*	Mean	*N*	Mean	*N*	Mean	*N*	Mean
Condoms	607	5	1,634	14	687	7	1,046	7
CoC pills	1,001	7	927	7	1,321	8	632	4
EC pills	389	4	38	3	537	5	291	3
Injections	1,104	7	894	7	1,666	10	983	7
IUCDs	501	4	6,291	46	581	4	6,315	41
Total	3,602	22	9,784	71	4,792	27	9,267	58

The pattern of products distributed was similar for the volume of clients (Table 3). MSS gave out more commodities per facility than DKT. The most common methods given by MSS were condoms and IUCD, while pills and injections were the main method for DKT. In the second period, DKT distributed fewer products per facility on average, while MSS increased its distribution.

**Table 3:** Volume of product given out on average to each client by facility type in the last month.

Service	2019–2020				2020–2021			
	DKT		MSS		DKT		MSS	
	*N*	Mean	*N*	Mean	*N*	Mean	*N*	Mean
Condoms*	1,151	13	6,970	64	1,548	18	8,092	54
CoC pills*	4,043	28	1,161	9	2,071	14	1,358	9
EC pills	1,012	9	14	14	1,279	13	590	5
Injections	1,516	11	796	6	1,654	11	954	6
IUCDs	1,344	12	6,016	44	911	8	6,530	42
Total	9,066	56	14,957	108	7,463	42	17,524	110

* Condoms were dispensed (and reported here) as packs of 3, CoC are reported as monthly strips.

Since each facility did not serve and distribute each type of client and product in both phases, the denominator was different for each method (Tables 7, 8 in Appendix). For the total, we used the total number of facilities in each phase (Table 1).

We estimated the average amount of products received by a client during their visit (Table 4). DKT clients received 2 packs of condoms, while MSS clients received 4 packs. DKT providers reported giving 1 injection and 3 IUCDs per visit to their clients (phase 1). During the COVID-19 period (phase 2), MSS providers reported giving twice as many condoms and pills as compared to phase one. In contrast, DKT providers gave out the same or slightly fewer commodities per visit during phase two. It is unclear why there are 2 or 3 IUCD listed per client visit by DKT providers.

**Table 4:** Product per client by facility type in the last month.

Service	2019–2020			2020–2021		
	DKT	MSS	Combined	DKT	MSS	Combined
	Mean	Mean	Mean	Mean	Mean	Mean
Condoms	2	4	4	2	8	6
CoC pills	4	1	3	2	2	2
EC pills	3	0.4	2	2	2	2
Injections	1	1	1	1	1	1
IUCDs	3	1	1	2	1	1
Total	3	2	2	2	2	2

Table 5 presents the results of a regression analysis for predictors of the volume of clients at facilities. After controlling for all other factors in the model, MSS had 3.15 times more clients than DKT facilities during phase 1 (IRR: 3.15; 95% CI: 2.74, 3.61) and 2.11 times more during phase 2 (IRR: 2.11; 95% CI: 1.79, 2.49). Absence of private space to talk about FP during a clinic visit was a key detractor of client visits that decreased client volume by 26% (IRR: 0.74; 95% CI: 0.67, 0.82). Factors that had no significant impact on client volumes were rural vs. urban location, the presence of an NGO logo on clinic board, work experience or duration of operation of the clinic, availability of IEC material or a washroom in the clinic. There was no difference observed in client volume during the COVID-19 period (2020–2021).

**Table 5:** Negative binomial regression for volume of clients visiting facilities.

Volume of Clients	Phase 1	Phase 2	Combined IRR (95% CI)
	2019–2020 IRR (95% CI)	2020–2021 IRR (95% CI)	
Facility type (26)			
MSS	3.15*	2.11*	2.49*
	(2.74, 3.61)	(1.79, 2.49)	(2.24, 2.79)
Region (Rural)			
Urban	1.09	0.86	0.95
	(0.94, 1.26)	(0.72, 1.02)	(0.84, 1.06)
Duration of operation of facility (Months)	1.00	1.000	0.999
	(0.99, 1.00)	(0.99, 1.001)	(0.999, 1.000)
Work experience	1.00	1.002	1.002*
	(0.99, 1.003)	(1.000, 1.004)	(1.000, 1.003)
Visible logo	0.89	0.83	0.84
	(0.67, 1.18)	(0.64, 1.08)	(0.69, 1.01)
Absence of privacy			
Overhearing conversations in counseling area	0.73*	0.75*	0.74*
	(0.64, 0.83)	(0.64, 0.87)	(0.67, 0.82)
Washroom	1.25	1.24	1.18
	(0.81, 1.93)	(0.72, 2.15)	(0.83, 1.68)
IEC material available			
Brochure	0.95	1.08	1.06
	(0.78, 1.14)	(0.85, 1.39)	(0.91, 1.24)
Booklet	0.93	1.21	1.06
	(0.81, 1.07)	(0.98, 1.49)	(0.94, 1.20)
Chart	0.86	0.84	0.88
	(0.70, 1.07)	(0.57, 1.23)	(0.72, 1.07)
COVID-19 (pre COVID-19)			
During COVID-19	–	–	0.98
			(0.88, 1.09)
Observations	**291**	**338**	**629**

* Statistical significance at 95% CI.

A regression for product distribution shows comparable results (Table 6). On average, MSS facilities gave out 2.3 times more products than DKT facilities (IRR: 2.26; 95% CI: 1.94, 2.63). As with volume of clients, there was no significant impact of rural vs. urban location, duration of operation or work experience, availability of IEC material or a washroom. However, visibility of the NGO logo increased product volumes by 69% only during phase 1 but had no impact in phase 2. Conversely, absence of privacy did not impact product volumes in phase 1 but reduced them by 22% in phase 2. There was 16% less product distributed during phase 2, while COVID-19 was in sway.

**Table 6:** Negative regression for volume of product.

Volume of products	Phase 1	Phase 2	Combined IRR (95% CI)
	2019–2020 IRR (95% CI)	2020–2021 IRR (95% CI)	
Facility type (26)			
MSS	1.89*	2.57*	2.26*
	(1.51, 2.38)	(2.09, 3.14)	(1.94, 2.63)
Region (Rural)			
Urban	1.05	0.95	0.97
	(0.83, 1.34)	(0.76, 1.18)	(0.83, 1.14)
Duration of operations of facility (Months)	0.99	0.99	0.99*
	(0.99, 1.001)	(0.99, 1.00)	(0.99, 1.00)
Work experience	1.002	1.004*	1.003*
	(0.99, 1.01)	(1.001, 1.006)	(1.001, 1.01)
Visible logo	1.69*	0.90	1.07
	(1.08, 2.67)	(0.65, 1.25)	(0.83, 1.42)
Invasion of privacy			
Overhearing conversations in the counseling area	0.88	0.78*	0.83*
	(0.71, 1.09)	(0.64, 0.95)	(0.72, 0.97)
Washroom	0.85	1.97	1.13
	(0.42, 1.70)	(0.99, 3.92)	(0.69, 1.85)
IEC material available			
Brochure	1.17	0.90	1.06
	(0.86, 1.59)	(0.65, 1.24)	(0.86, 1.31)
Booklet	0.93	1.41*	1.11
	(0.74, 1.17)	(1.08, 1.85)	(0.93, 1.32)
Chart	0.88	0.89	0.89
	(0.62, 1.24)	(0.55, 1.43)	(0.68, 1.19)
COVID-19 (pre COVID-19)			
During COVID-19	–	–	0.84*
			(0.72, 0.97)
Observations	**291**	**338**	**629**

* Statistical significance at 95% CI.

## Discussion

This is the first study to describe clinic volumes of major private providers of FP i.e., DKT and MSS in Pakistan. We found that MSS facilities consistently outperformed DKT facilities in both client volumes and product distribution across two phases. However, overall daily clientele is low for both NGOs. Common methods given out by MSS were condoms and IUCDs, while DKT served more clients with pills and injections. During COVID-19, MSS reported distributing short-term methods such as condoms and pills, whereas DKT's distribution remained static. Regression analysis revealed that MSS facilities had significantly more clients and distributed more products than DKT facilities. Absence of privacy was a major detractor for client volumes. Having an NGO logo, duration of practice, presence of a washroom or IEC materials did not affect client volumes. The COVID-19 pandemic led to a 16% reduction in product distribution during phase 2.

In Pakistan there is substantial underutilization of both private and public FP health facilities, underscoring significant room for scaling up client volumes at DKT and MSS supported clinics, other NGOs, and public sector clinics (29). DKT and MSS compare favorably with the public sector population welfare department facilities, which serve an average of two clients every three days, based on 725,000 clients served annually from its 3,300+ facilities nationwide (3). To address these gaps in FP services, it is crucial to enhance the capacity of existing clinics, such as the 300+ MSS clinics nationwide serving around 200,000–250,000 clients annually and 1,200+ DKT clinics nationwide serving around 250,000–300,000 clients annually. A previous analysis suggested that acquiring Pakistan's FP2030 targets requires a substantial increase in users, with an estimated additional 9 million users to achieve national targets (14). Mere tripling of these low volumes (to 5–9 clients a day) can add one million clients from these clinics alone, and more so if one includes the 7,000+ clinics supported by Greenstar Social Marketing and 125 by Rahnuma-Family Planning Association of Pakistan. Since a greater volume of clients increase efficiency and lowers costs per client, expanding the clientele would increase operating costs only marginally or not at all (30–32). In turn, these savings may eventually be used to open new efficient clinics and increase the total clientele of these NGOs (or of government facilities) in previously underserved areas (33).

### Service and product outreach of DKT and MSS

Both MSS and DKT offer a wide array of method choices, reflecting the mandate of the DAFPAK project, and is consistent with the concept that availability of multiple methods increases overall uptake of FP (34). However, the specific method-mix also depends on the core model of the organization. IUCD and condoms for MSS, along with sterilization surgeries that are not part of the DAFPAK package and middle and high-end contraceptive pills and condoms for DKT. During COVID-19, as client volumes shrank, MSS clients received more condoms per visit, presumably to minimize contact, while the overall pattern remained unchanged for DKT. The high number of IUCD (2–3 per client) dispensed by DKT providers is unexplained.

### Factors that affect clientele

Client satisfaction is considered as an indicator of quality service provision (35). Several factors affect client volumes including branding (particularly for socially franchised facilities), training and experience of providers, more spacious clinics, availability of washrooms, IEC material and appropriate privacy (19, 26). We found that a major factor associated with high client volumes is a facility's reputation for providing privacy. Prioritizing privacy and confidentiality are shown to be protective in reducing the discontinuation of family planning methods (35). Many health and family planning facilities are small establishments with 1–2 rooms, where patients are sometimes examined and counseled in the same room where other clients are also waiting, or in other instances, a mere curtain separates the two. It is therefore a major concern, particularly in communities where FP is not discussed publicly (35, 36).

Beyond privacy, studies have shown that providers' work experience, availability of equipment, and counseling material are significantly associated with client satisfaction and visits to FP facilities (26, 37). The fact that our analysis showed no association of these factors with client visits or products dispensed raises interesting questions about the role perceived quality plays in driving foot traffic at health facilities, and may be studied in future work. The presence of a banner displaying the name of the supporting organization was complex. While displaying such a banner did not affect client volumes, it increased the amount of product taken by clients per visit, suggesting that presence of a banner may increase trust, which in turn leads to higher quantities of contraceptives being purchased.

### COVID-19 and family planning through social franchising

Many low and middle-income countries experienced a decline in method uptake, higher discontinuation of methods and fewer facilities visits during COVID-19 (38). A World Health Organization survey revealed that almost 70% of 105 countries experienced disruptions in family planning services due to the pandemic (39). These were driven by demand factors such as fear of acquiring infection at clinic visits, lack of knowledge about contraceptive availability during COVID-19 times, preferences for methods requiring with no or fewer trips to health facilities, restricted access to family planning facilities (24). Demand factors that further limited FP uptake were fewer clinics open, reduced outreach or referrals, limited counselling regarding side effects of contraceptives, and stock-outs related local or global supply chain disruption due to COVID-19 (32, 33). Specific changes in method mix varied across countries. A shift to condoms seen in our study is consistent with United Kingdom (40) and Ghana (41) where clients switched to short-term methods rather than long-term methods and may have been related to greater mobility of men, which may have been accentuated during COVID-19 (42). Moreover, condoms do not require medical personal assistance or counselling, hence, increasing its uptake (43). However, around 10% of women in Kenya and Burkina Faso shifted to more effective methods like injections and IUCDs that also minimized contact between clients and providers (44).

Providers in DAFPAK adapted by implementing social distancing and personal protection guidelines. Our results show that both the number of client visits and product uptake decreased during COVID-19. The average client visit per facility decreased for each method except EC pills and injections, as did the average product given out per facility, with the highest decreases for CoC pills and the least for emergency contraception pills and injections. Between the two-time periods, the product mix shifted from CoC pills (22%–14%) to mainly condoms (34%–39%) followed by EC pills (4%–7%) during the COVID-19, while injections (10%) and IUCDs (31%) remained unchanged. In essence, there was a slight shift from more reliable methods to condoms and presumably some dropouts.

### Limitations

Our study is limited by the fact that it depicts data from only two private sector providers, DKT and MSS. Although they are among the largest of such NGOs, the absence of Greenstar Social Marketing (GSM), the Family Planning Association of Pakistan (FPAP) and outreach NGOs is a gap. While utilization patterns of those NGOs is likely similar (Dr. Aziz Rab, CEO GSM, personal communications), it would have been useful to formally document their patterns. Secondly, since private providers don't often keep service records, data were collected using supply lists plus some element of recall which could have added errors.

### Recommendations

To enhance family planning services, prioritize enhancing the serving capacity of existing DKT and MSS clinics over opening new clinics, reducing costs per user and improving clinic efficiency. The cost savings can then be used to open new, efficient clinics in previously underserved areas, expanding the clientele. Improve the physical infrastructure of health and family planning facilities, particularly in low-resource settings, ensuring better privacy and confidentiality for clients by providing more spacious clinics, and separate rooms for counseling/examinations and waiting. Additionally, investing in providing training on privacy and confidentiality could enhance client satisfaction, increasing family planning method uptake and continuation. Additionally, enhance access to family planning services during pandemics by promoting telemedicine and home delivery services for contraceptives, mitigating disruptions caused by factors such as fear of infection, reduced clinic visits, and supply chain disruptions, ensuring continuous access to contraceptives and supporting method continuation.

## Conclusion

This study provides insight into the effectiveness of private-sector providers. The private sector has better utilization but a smaller footprint than the public sector. However, both public and private sector health and FP facilities are significantly underutilized and can improve substantially. In particular, private (mostly urban) and public (more often rural) clinics are complementary in where they are located and the clientele they serve. Thus, such contributions from the private sector are vital if Pakistan were to achieve its FP2030 goals. Moreover, while FP services are enhanced, COVID-19 highlighted the importance of emergency preparedness to avoid crucial service outages in emergencies. This would include both the means to offer services and to maintain supply chains to ensure appropriate commodity security is assured.

## Data Availability

The raw data supporting the conclusions of this article will be made available by the authors, without undue reservation.

## Appendix

**Table 7:** Facilities providing services (clients) across both phase.

Service	2019–2020			2020–2021		
	DKT (163)	MSS (138)	Total (301)	DKT (178)	MSS (160)	Total (338)
Condoms	112	113	225	100	147	247
CoC pills	144	127	271	165	148	313
EC pills	103	13	116	110	94	204
Injections	155	132	287	172	151	323
IUCDs	120	138	258	135	154	289

**Table 8:** Facilities providing products across both phase.

Service	2019–2020			2020–2021		
	DKT (163)	MSS (138)	Total (301)	DKT (178)	MSS (160)	Total (338)
Condoms	86	109	195	88	150	238
CoC pills	145	127	272	145	150	295
EC pills	114	1	115	101	109	210
Injections	139	125	264	151	150	301
IUCDs	113	137	250	111	157	268
